# Novel hepatoviruses in synanthropic bats in the upper Midwestern United States

**DOI:** 10.1007/s00705-022-05610-8

**Published:** 2022-09-22

**Authors:** Gun Temeeyasen, Ben M. Hause

**Affiliations:** Department of Veterinary and Biomedical Sciences and Animal Disease Research and Diagnostic Laboratory, South Dakota State University, Brookings, SD 57007 USA

## Abstract

A nearly complete genome sequence of hepatovirus G was isolated from an *Eptesicus fuscus* bat submitted for rabies virus testing due to human exposure in South Dakota. The predicted polyprotein sequence was 78.2% and 74.4% identical to genotypes G1 and G2, respectively, recovered from bats in Ghana. Quantitative PCR on 90 *E. fuscus* bats showed that eight (8.9%) were positive for hepatovirus G. Targeted sequencing of the VP2 region of the genome for five positive samples showed >99% identity to hepatovirus G strain Ef15893, demonstrating that hepatovirus G commonly circulates in *E. fuscus* bats in the upper Midwest.

**Supplementary Information:**

The online version contains supplementary material available at 10.1007/s00705-022-05610-8.

Pandemics originating from zoonoses have highlighted the need for surveillance of viruses circulating in wildlife. Bats are important reservoirs of diverse viruses, many of which have spilled over to humans [1, 2]. Prominent examples include severe acute respiratory syndrome coronavirus (SARS-CoV), Ebola virus, Nipah virus, Hendra virus, and, likely, SARS-CoV-2 [3]. Rabies virus, one of the oldest viruses known to humans, resides in a bat reservoir [4, 5]. Infection of humans with rabies virus is always fatal without medical intervention, necessitating testing of bats with human exposure.

Here, metagenomic sequencing was performed on an *Eptesicus fuscus* bat submitted for rabies virus detection due to human exposure. No specific institutional animal care and use committee approval was required, as the bat specimens were submitted for diagnostic testing. The bat tested negative for rabies virus by direct fluorescent antibody testing. As part of an ongoing project to characterize viruses present in synanthropic bats, metagenomic sequencing was performed on a pooled viscera homogenate from organs of the abdominal and thoracic cavities. The homogenate was clarified by centrifugation at 14,000 x *g* for 5 minutes, followed by digestion with a nuclease cocktail to degrade unprotected DNA and RNA [6–8]. Next, DNA and RNA were isolated using a QIAamp^®^ Viral RNA Mini Kit (QIAGEN, Hilden, Germany). Polyadenylated RNA was further purified using Dynabeads™ Oligo (dT)_25_ (Thermo Fisher Scientific, Vilnius, Lithuania) prior to reverse transcription using barcoded random hexamers FR26RV-N [9]. Following second-strand synthesis using Sequenase version 2.0 DNA polymerase (Thermo Fisher Scientific), DNA was amplified by PCR using the barcode primer FR20RV [9]. Sequencing libraries were prepared from purified amplicons using a Nextera XT DNA Library Preparation Kit (Illumina, San Diego, CA, USA) and sequenced on a MiSeq system using paired 151-base-pair reads. A total of 433,538 reads were generated.

Next-generation sequencing reads were assembled *de novo* using CLC Genomics version 21. Assembled contigs were identified by BLASTx using the Cloudblast feature in Omicsbox version 2.0.36. A 6,921-nucleotide (nt) contig comprised of over 116,000 reads was identified with 78.2% identity to the sequence of a hepatovirus G genome recovered from a *Coleura afra* bat in Ghana (GenBank no. NC038316). The average genome coverage was 2,371-fold. Open reading frame analysis identified a predicted 2,153-amino-acid protein derived from a genome with a GC content of 37.5%. The 392-nt 5’ and 67-nt 3’ untranslated regions were incomplete based on comparisons to NC038316, with 5’ and 3’ untranslated region sequences of 723 and 78 nt. The nearly complete genome sequence of hepatovirus G strain Ef15893 was submitted to the GenBank database under accession no. OM302498. Mammalian orthoreovirus and Eptesicus bat coronavirus were also detected in the tissue homogenate; however, limited sequence reads prohibited further genetic analysis.

Phylogenetic analysis was performed on the polyprotein sequence to determine the evolutionary relationship of hepatovirus G Ef15893 to other members of the genus *Hepatovirus*. Polyprotein sequences were aligned using ClustalW [10], and the phylogeny was reconstructed by the maximum-likelihood method, using the LG+G+F model. Hepatovirus G strain 15893 was located in a monophyletic clade comprised of hepatovirus G1 and G2, which formed a sister clade to hepatovirus H (Fig. 1). Interestingly, the three genotypes of hepatovirus H were obtained from rodents and bats, suggesting that closely related hepatoviruses have the ability to infect multiple species.

**Fig. 1:**
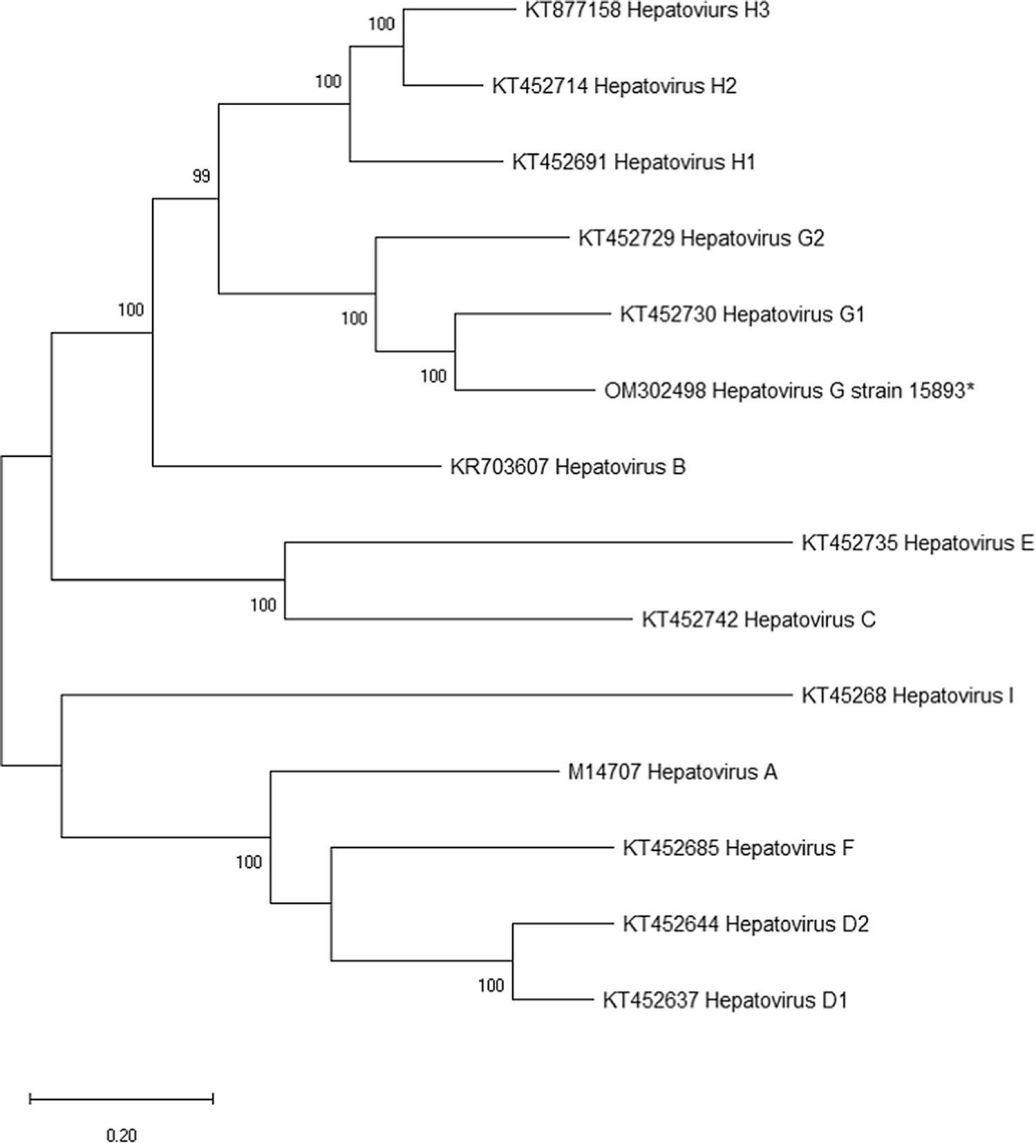
Phylogenetic analysis of polyprotein sequences of members of the genus *Hepatovirus* performed in MEGA X using the maximum-likelihood method [3]. Bootstrap values were calculated from 1,000 replicates, and values greater than 70 are shown. Sequences determined here are indicated by “*”.

To estimate the prevalence of Ef15893 in Midwestern bats, a 5’-nuclease assay was designed targeting the RNA-dependent RNA polymerase region of the genome, using the following oligonucleotides: forward, 5’-TTCTGAAGGACAAAGTAGGGC; reverse, 5’-TCAAAAGGCTGGTACAAGGG; probe, 5’-FAM-TCCAGATGGCATAGAACCCGACAC. Quantitative reverse transcription PCR was performed on RNA isolated from a viscera homogenate prepared from 90 *E. fuscus* bats submitted for rabies virus testing from South Dakota and surrounding states. Eight samples (8.9%) were positive, with cycle threshold values of 29.5 to 36.9. A region of VP2 was amplified by PCR for five positive samples and sequenced. No amplicon was generated from the remaining three samples, all of which had C_t_ values ≥33.9. The sequences of all five samples were greater than 99% identical to Ef15893. Together, these results suggest that hepatovirus G infections are common in *E. fuscus* in the upper Midwest. A recent paper also reported detection of hepatovirus G sequences in bat guano collected from a California roost dominated by *Corynorhinus townsendii*, further suggesting that hepatovirus G is widespread in U.S. bats [11].

Hepatitis A virus is the best-characterized member of the genus *Hepatovirus*. Hepatitis A virus infects humans and other primates and causes acute hepatitis, resulting in an estimated 11,000 deaths annually [12]. Human infections occur through ingestion of contaminated material and are not zoonotic in origin. Despite a lack of evidence for human infections with other non-primate hepatoviruses, bat antisera were able to bind and neutralize HAV, suggesting conservation of virus antigenicity [13]. Members of nine species of hepatoviruses have been identified in diverse mammals, many of which are synanthropic [14]. A recently discovered hepatovirus in goats further expands the known host range of hepatoviruses from wildlife to livestock [15]. Further research is warranted to identify and characterize wildlife viruses that may pose a risk of zoonosis.

## Supplementary Information

Below is the link to the electronic supplementary material.

Supplementary file1 (TXT 13 KB)

## Data Availability

The genome sequence of hepatovirus G strain Ef15893 was submitted to the GenBank database under accession OM302498. Metagenomic sequencing reads are available as Bioproject PRJNA798416.
